# Establishing Content Validity for the Nutrition Literacy Assessment Instrument

**DOI:** 10.5888/pcd10.120267

**Published:** 2013-07-03

**Authors:** Heather Gibbs, Karen Chapman-Novakofski

**Affiliations:** Author Affiliation: Karen Chapman-Novakofski, University of Illinois, Urbana, Illinois.

## Abstract

**Introduction:**

Identification of low levels of health literacy is important for effective communication between providers and clients. Assessment instruments for general health literacy are inadequate for use in nutrition education encounters because they do not identify nutrition literacy. The primary objective of this 2-part study was to assess content validity for the Nutrition Literacy Assessment Instrument (NLAI).

**Methods:**

This study included a 35-item online survey of registered dietitians (134 of whom answered all questions) and a pilot study in which 5 registered dietitians used the NLAI among 26 clients during nutrition education consultations. To assess agreement with the NLAI by survey participants, we used the following scale: “necessary” (70% agreement), “adequate” (80% agreement), or “good” (90% agreement); comments were analyzed by using content analysis. For the pilot, we made comparisons between subjective assessments, the Rapid Estimate of Adult Literacy in Medicine (REALM), and the NLAI. Registered dietitians also completed a postpilot–study survey.

**Results:**

For the online survey, we found good agreement (average, 89.7%) for including each section of the NLAI. All sections accomplished their purpose (average, 81.5%). For the pilot, REALM and NLAI correlation (*r* = 0.38) was not significant; the subjective assessment of clients by dietitians and NLAI lacked agreement 44% of the time, and registered dietitians provided instruction on deficient knowledge and skills identified by the NLAI 90% of the time.

**Conclusion:**

The NLAI is a content-valid measure of nutrition literacy. Additional validation of the NLAI is important because an objective instrument is needed for identifying nutrition literacy, a construct that appears to be different from health literacy.

## Introduction

Health literacy is “the degree to which individuals have the capacity to obtain, process, and understand basic health information and services needed to make appropriate health decisions” (1). Assessment instruments (2–4) provide guidance for choosing patient health information according to individual health literacy needs.

Nutrition is important for disease prevention, but increasing rates of chronic diseases suggest a need for nutrition education. However, nutrition information is complex and may require high levels of cognitive skills (5). Most registered dietitians do not assess health literacy, perhaps because current instruments do not assess nutrition literacy. Nutrition literacy requires knowledge of nutrition principles and skill in food-related tasks (6).

The Rapid Estimate of Adult Literacy in Medicine (REALM) is a valid and reliable instrument that predicts reading ability of health care–related words (2). Two instruments relate to nutrition literacy assessment: the Newest Vital Sign (7) and the Nutrition Literacy Scale (8). The food label used in the Newest Vital Sign purports to measure numeracy, but this instrument does not assess nutrition knowledge. The Nutrition Literacy Scale attempts to measure comprehension of nutritional information, but it is unclear whether it provides any measure beyond print literacy, and other uses have not been described in the literature.

Consequently, we developed the Nutrition Literacy Assessment Instrument (NLAI). Literature review and expert interviews (6) were the basis for including the following domains: appreciation of relationships between nutrition and health, knowledge of macronutrients, food measurement skill, numeracy and label reading, and skill in grouping like foods.

The objectives of this 2-part study were to 1) establish content validity of the NLAI and identify attitudes toward needs and constructs for a nutrition literacy instrument (survey) and 2) test the usability of the instrument in nutrition education settings and determine its impact on teaching delivery from the registered dietitian’s perspective (pilot).

## Methods

### Survey: critique of instrument

A 35-item survey was designed to assess content validity for the NLAI and attitudes among dietitians toward nutrition literacy. Content validity reflects the relevance of the assessment instrument to its targeted construct (9) and usually requires expert review (10). Content validity should be determined before construct validity, which is the accuracy with which the assessment measures the targeted construct. The survey had 7 sections; the first section addressed whether survey participants found the instrument’s algorithm useful and understandable. For the next 5 sections of the survey (nutrition and health, macronutrients, household food measurement, food labels and numeracy, and food groups), participants were asked whether the instrument accomplished its purpose, whether the questions were appropriate in difficulty, whether anything important had been left out, and whether the section was important to include. For the section on nutrition and health, a question on length replaced a question on whether anything was left out. Each question of the survey allowed participants to provide additional comment. The seventh section addressed health literacy, and because another research question was whether dietitians would prefer to use the REALM instead of the NLAI, we asked 2 questions about the REALM. 

All methods were approved by the University of Illinois institutional review board (IRB). Two dietetic practice groups (each with approximately 6,400 members) of the Academy of Nutrition and Dietetics (formerly the American Dietetic Association) were selected as participants in the survey because their practice areas involve nutrition education. Methods were approved by the dietetic practice groups and the Academy of Nutrition and Dietetics. All data were collected in December 2011.

The survey recruitment invitation was distributed by an e-mail that included a link to the survey, which was designed by using online survey software (SurveyGizmo, Boulder, Colorado). Data were automatically saved as an Excel (Microsoft Corp, Redmond, Washington) spreadsheet. Of 385 potential participants, 377 (98%) consented to the survey, 178 (46%) answered some questions, and 134 (35%) completed the entire survey. Of the 243 who consented but did not finish, 211 left the survey immediately after consent; this drop-off might be explained by the temporary web-browser incompatibilities on the day the survey was released. 

For statistical analyses, we used PASW Statistics for Windows, version 18.0 (SPSS Inc, Chicago, Illinois). To evaluate the degree of agreement with the NLAI by survey participants, we compared our data with the following scale: agreement at or above 70% is necessary, agreement at or above 80% is adequate, and at or above 90% is good (11). Additionally, we analyzed comments by using content analysis, which involves identifying coherent and important examples, themes, and patterns (12). Two researchers analyzed the comments separately to develop a list of keywords and codes and assigned comments to categories. Then each reviewed the results of the other; they agreed on assignment of comments to categories for 363 of 377 (96%) comments and discussed differences until consensus was reached. Overarching themes were developed from the codes that had the highest frequency of similar response.

### Pilot study

Five registered dietitians were recruited from personal contacts for the pilot study; because they were viewed as coinvestigators, they completed human subjects training before participation. They were recruited from 4 participating sites: a regional bariatric surgical facility, 2 metropolitan outpatient clinics, and a private practice. The pilot study was implemented in the metropolitan areas of Chicago and Urbana-Champaign, Illinois. All methods received IRB approval, and all data were collected between May 2011 and May 2012.

Participants in the pilot study were trained by the first author (H.G.) on health literacy so they would know how to interpret and act upon the results of the NLAI. Health care providers who receive training on health literacy have greater intentions of identifying patients with low levels of health literacy and of checking for patient understanding of information provided (13) and more often use methods and educational materials designed for people who have low levels of health literacy (14). The training included 3 online educational modules, a packet of research materials, and a telephone conversation with the researchers to clarify the research process.

Adult clients (aged >18) were recruited through a flyer by clinic personnel using a convenience sample approach at selected outpatient clinics in the dietitians’ normal clinic area. We targeted 10 completed surveys per participating dietitian within 1 month. If the dietitian determined the client was not suitable because of cognitive impairment or an inability to read, the patient did not participate. A prescreening instrument was required by the IRB at 1 site to ensure competency for participation.

After consent forms were completed, the dietitian completed a subjective assessment (a short form to indicate whether the dietitian’s initial impression, based on general observation, of the client’s nutrition literacy was adequate, marginal, or inadequate); read the instructions for the REALM and administered the interview; gave the NLAI to the client to complete, and recorded the time required to complete the NLAI. The REALM is scored by totaling the number of words (of 66) read correctly, yielding a predicted age-level reading. On the basis of the number of correct answers, each section of the NLAI is scored as “suggests high likelihood of inadequate nutrition literacy,” “suggests marginal nutrition literacy,” or “suggests adequate nutrition literacy.” The dietitian then proceeded with the scheduled nutrition education and completed a postpilot–study survey. We used nonparametric correlation tests (Spearman ρ) to evaluate the relationship between the NLAI and the REALM.

## Results

### Survey: critique of instrument

We found that the algorithm achieved adequate agreement (81.5%) on the basis of yes answers to “Is the algorithm easy to understand and follow?” (93.1%) and “Is this section important to include?” (83.1%) and no answers to “Are there decisions that are missing?” (68.1%) (Table 1). Almost one-third (29.5%) of participants believed decisions had been left out of the algorithm; the most common themes identified as being left out were “language/cognitive barriers” (14 comments), “readiness to learn” (5 comments) and “ability to purchase and prepare food” (4 comments).

**Table 1:** Results of Survey Among Registered Dietitians on Nutrition Literacy Assessment Instrument (NLAI), 2011

Question	No. of Responses	n (%)	n (%)	n (%)
**Algorithm**		**Yes**	**No**	**Neither^a^**
Does the algorithm accomplish its purpose?	178	153 (85.9)^b^	16 (8.9)	9 (5.0)
Is the algorithm easy to understand and follow?	176	164 (93.1)^c^	9 (5.1)	3 (1.7)
Is this section important to include?	166	138 (83.1)^b^	22 (13.2)	6 (3.6)
Are there decisions that are missing?	165	48 (29.5)	111 (68.1)	6 (2.5)
**Nutrition and health**		**Yes**	**No**	**Neither^a^**
Does this section accomplish the purpose of measuring reading comprehension?	157	131 (83.4)^b^	22 (14.0)	4 (2.6)
Does this section accomplish the purpose of identifying client’s understanding of relationship between nutrition and health?	157	133 (84.7)^b^	16 (10.2)	8 (5.1)
Is this section important to include in the instrument?	157	127 (80.9)^b^	23 (14.6)	7 (4.5)
		**Yes**	**No, Too Short**	**No, Too Long**
Is the passage appropriate in length?	157	105 (66.9)	3 (1.9)	49 (31.2)
		**Yes**	**No, Too Easy**	**No, Too Hard**
Are the questions appropriate in difficulty?	155	109 (70.3)	15 (9.7)	31 (20.0)
**Macronutrients**		**Yes**	**No**	**Neither^a^**
Does this section accomplish the purpose of identifying knowledge of macronutrients?	149	133 (89.3)^b^	14 (9.4)	2 (1.3)
Is this section important to include in the instrument?	147	128 (87.1)^b^	17 (11.6)	2 (1.4)
Has anything been left out of this section that you feel is important?	148	32 (21.6)	114 (77.0)	2 (1.4)
		**Yes**	**No, Too Easy**	**No, Too Hard**
Are the questions appropriate in difficulty?	146	101 (69.2)	1 (0.7)	44 (30.1)
**Household food measurement**		**Yes**	**No**	**Neither^a^**
Does this section accomplish the purpose of identifying ability to estimate portion size?	145	122 (84.1)^b^	22 (15.2)	1 (0.7)
Is this section important to include in the instrument?	147	140 (95.2)^c^	6 (4.1)	1 (0.7)
Has anything been left out of this section that you feel is important?	148	60 (40.5)	88 (59.5)	NA
		**Yes**	**No, Too Easy**	**No, Too Hard**
Are the questions appropriate in difficulty?	147	143 (97.3)^c^	0	4 (2.7)
**Food label and numeracy**		**Yes**	**No**	**Neither^a^**
Does this section accomplish the purpose of identifying skill with use of food labels?	146	139 (95.2)^c^	6 (4.1)	1 (0.7)
Is this section important to include in the instrument?	145	137 (94.5)^c^	8 (5.5)	0
Has anything been left out of this section that you feel is important?	142	26 (18.3)	103 (72.5)	13 (9.2)
		**Yes**	**No, Too Easy**	**No, Too Hard**
Are the questions appropriate in difficulty?	146	107 (73.3)	0	39 (26.7)
**Food groups**		**Yes**	**No**	**Neither^a^**
Does this section accomplish the purpose of identifying ability to group foods?	141	139 (98.6)^c^	2 (1.4)	0
Is this activity appropriate in difficulty?	139	135 (97.1)^c^	4 (2.9)	0
Is this section important to include in the instrument?	140	127 (90.7)^c^	13 (9.3)	0
Has anything been left out of this section that you feel is important?	139	23 (16.5)	112 (80.6)^b^	4 (2.9)
**Health literacy**				
Would you prefer to use the REALM instead of the NLAI to assess nutrition literacy?	139	20 (14.4)	111 (79.9)	8 (5.8)
Are there any sections of the REALM that you feel would be beneficial to include on a new assessment tool?	134	34 (25.4)	98 (73.1)	2 (1.5)
Do you use an instrument to assess health literacy in your clients?	140	10 (7.1)	130 (92.9)	NA
Would you use an instrument if there was one available?	134	98 (73.1)	28 (20.9)	8 (6.0)
Is health literacy an issue that you feel is important?	138	133 (96.4)	5 (3.6)	NA
Is an assessment of nutrition literacy important enough to nutrition education to take the time for an assessment?	139	112 (80.6)	25 (18.0)	2 (1.4)

Abbreviation: REALM, Rapid Estimate of Adult Literacy in Medicine; NA, not applicable.

a Category added by the researchers after the survey was administered for participants who did not choose yes or no but commented in a way that was not distinguishable as yes or no.

b Answer had adequate agreement (>80%).

c Answer had good agreement (>90%).

For yes answers to the question, “Is this section important to include in the instrument?” the sections achieving adequate agreement were nutrition and health (80.9%) and macronutrients (87.1%), whereas the sections achieving good agreement were household food measurement (95.2%), food label and numeracy (94.5%), and food groups (90.7%). With an overall score of 89.7%, these 5 sections achieved good agreement for importance.

Combining yes answers to whether the section accomplished its purpose and whether the section was appropriate in difficulty or in length and no answers to whether anything had been left out, we found that 5 sections achieved agreement. The sections on nutrition and health (76.3%) and macronutrients (78.5%) achieved the minimum required for agreement; the sections on household food measurement (80.3%) and food label and numeracy (80.3%) achieved adequate agreement; and food groups (92.1%) achieved good agreement.

For the section on nutrition and health, 15 participants commented that the reading level was too high, 13 commented that the section was too long or wordy, and 9 commented that the concepts were too advanced. For the section on macronutrients, 10 participants commented that the section was too difficult or “encourages guessing.” For the section on household food measurement, 19 participants commented that visual references in pictures are needed for better size estimation; 11 noted issues with the use of the word “portions”; 8 suggested modifying the “milk image”; and 39 made suggestions for including various alternative foods. 

The section on food label and numeracy achieved a high score for importance (94.5%) and purpose (95.2%), but it did not score as well for the questions on whether anything had been left out (only 72.5% said no) and difficulty (26.7% said section was too hard). Fifteen participants commented that 1 question, which requires computation of percentages, is too hard, and 8 commented the section overall is too difficult.

Most participants agreed the section on food groups is important (90.7%), achieved its purpose (98.6%), and left nothing important out (80.6%). Six participants noted a need for an “others” category, which might include foods such as added sugars or oils, and 4 noted that a “combination food” (foods that are composed of more than 1 food group, such as tacos or lasagna) should be added as food categories.

Most (79.9%) participants did not prefer to use the REALM instead of the NLAI to assess nutrition literacy. Whereas 92.9% of participants indicated they do not use health literacy assessment instruments, 73.1% agreed that if an instrument designed for nutrition was available, they would use it in their practice. Almost all participants (96.4%) agreed that health literacy is important, and most (80.6%) agreed that an assessment of nutrition literacy is worth the time it would require. Of the 98 participants who indicated they would use an instrument if one was available, 10 indicated yes, if time is available. Of those who said they would not used such an instrument, 5 participants indicated there is not enough time to use an instrument during nutrition education encounters, and 4 participants said they prefer an interactive approach.

### Pilot study

Twenty-five clients completed the REALM, and 26 clients completed the NLAI (Table 2). Most (n = 23) scored greater than 61 on the REALM (predictive of a reading level above 9th grade), but of those, 7 clients scored at marginal nutrition literacy and 1 client scored at inadequate nutrition literacy for at least 1 area of the NLAI. The Spearman ρ correlation between the REALM and NLAI was not significant (*r* = 0.38; *P* = .06). Additionally, the subjective assessment and the NLAI indicated lack of agreement at a rate of 44%. For the 10 clients who received less than adequate scores on the NLAI, the dietitian provided instruction on the deficient knowledge or skill area 90% of the time. All 5 dietitians indicated that the time needed to complete the assessment is “about right” (Table 3). 

**Table 2:** REALM and NLAI Scores for Nutrition Education Clients Participating in Pilot Study on Nutrition Literacy, 2011–2012

Category	No. of Clients Evaluated	Mean (SD)	Range
REALM score^a^	25	63.9 (3.8)	50–66
Time it took to complete NLAI, min	20	8.4 (3.0)	4–15
NLAI score^b^, % correct	26	86.8 (9.6)	58.3–100.0
Readability of educational materials, grade level^c^	23	7.0 (1.1)	3.0–7.6

Abbreviations: REALM, Rapid Estimate of Adult Literacy in Medicine; NLAI, Nutrition Literacy Assessment Instrument.

a Based on maximum score of 66.

b Although the maximum possible score on the NLAI is 40, some clients were not instructed to complete the entire instrument; thus, we converted scores to percentage correct.

c Readability assessed by Flesh-Kincaid Grade Level in Microsoft Word 2010 (Microsoft Corp, Redmond, Washington).

**Table 3:** Postpilot Survey Among Registered Dietitians (n = 5) on Nutrition Literacy Assessment Instrument, 2011–2012

Question	No. of Responses
**The time needed to complete the assessment is . . .**	
About right	5
Too long	0
**Was the content of the assessment applicable to the needs of the client?**	
Yes	5
No	0
**Does the instrument adequately separate clients into different levels of understanding?**	
Yes	3
No	1
Not applicable	1
Comment	“I felt that with the very few clients that I tested that sometimes reading level and understanding don’t necessarily coincide. One lady did well on the test but had a more limited reading ability.”
**Please rank the difficulty experienced by clients in completing the assessment.**	
Very difficult	0
Difficult	0
Appropriate	4
Too easy	1
**Do you have any suggestions for improvements?**	“It was perceived that some of the clients did not want to take the time to complete the assessment, and others may have been too intimidated to participate. My only suggestion would be to provide an incentive for participation. Possibly a handout or booklet on improving health literacy.”

## Discussion

The NLAI was valid as tested within this study, and dietitians preferred the NLAI over the REALM. However, nutrition literacy is a complex concept, and responses to some questions deserve further investigation after the instrument is refined. In addition, the methods used to evaluate nutrition literacy need to reflect this complexity. “Dimensionality . . . the number of theoretically and empirically distinct subcomponents of the broader construct” (15) is important to the design of nutrition instruments because gaining an understanding of one’s nutrition literacy is dependent upon the complex interplay of knowledge of related subtopics as reflected in the NLAI. 

Several sections of the NLAI require further investigation. For instance, the responses using the cloze method for the section on nutrition and health indicated that the section accomplished its purpose but may be too long and difficult. Although others have used simpler and shorter assessment techniques (15), these techniques may not capture the essence of the questions’ rationale. Diet and health is complex, and requires a higher order of integrated conceptualization than perhaps multiple response items can divulge. However, the cloze method has been used in assessing the understanding of other health-related topics, such as prostate cancer (16), pharmacy instructions (17), and cardiovascular disease risk (18). Therefore, while the cloze method is used in health arenas other than nutrition, dietitians may be unfamiliar with, uncomfortable with, or lack knowledge about the applicability of this method.

Our approach to the section on macronutrients is similar to the approach used for the 23-item Nutrition Knowledge test (15), which asks participants to identify the nutrient content of foods. Ideally, an instrument should stratify participants into categories of nutrition literacy, requiring questions with varying levels of difficulty. Deciphering which questions to include or exclude according to difficulty is necessary and is determined by measures of construct validity. Because the section on macronutrients has not been widely evaluated with clients, the range in difficulty of questions or appropriate foods to include cannot be determined at this time.

Food photographs (like the photographs used for the food portions section of the NLAI) are a useful aid for estimating food portions (19) and have been studied with varying degrees of success for use in reporting portion sizes in food consumption studies (20–22). It is not known, however, whether food photographs labeled by common household measurements (such as those in the NLAI) improve accuracy of estimation (23). Our questions did not ask participants to estimate the amount seen in photographs because the amounts were identified in the questions. Rather, participants were asked to identify whether the stated amount for a given food is the “right” portion. In our study, the photographs served as a visual cue for proportionality, but they may not be necessary if knowledge of common food measurements is strong.

The section on food label and numeracy was adapted from the Newest Vital Sign, a reliable and valid measure of health literacy. Because it uses a nutrition label as the text reference, it is not on its own a measure of nutrition literacy but rather a measure of the ability to read food labels. Food labeling is an area with which the public has struggled — to the point where front-of-package labeling and healthfulness scores have been suggested (24). Another food label literacy tool has been developed for children; it uses 10 items to determine children’s ability to make healthful choices based on the Nutrition Facts label. Each item presents 2 labels and asks which choice is the more healthful (25). Future developments of the NLAI could consider similar items for an adult version.

Conceptually, food groups have been central to nutrition education since 1916 (26), so we were not surprised that our survey participants indicated food groups are important to evaluating nutrition literacy. Several participants commented that combination foods should be addressed in the NLAI; however, the US Department of Agriculture’s MyPlate food guidance graphic does not incorporate combination foods, nor did previous food guides. We question whether the general public recognizes that some foods can be classified into more than 1 food group. In addition, the concept of food groups may be different for the client than for the professional. For instance, a study among low-income African American women found the names of food groups may differ between the women and the professionals, as well as foods attributed to the food groups (27).

This study had several limitations. Dietitians were unable to recruit 10 clients within 1 month at any of the participating sites. Through personal communication, the dietitians provided the following reasons for this inability: summer vacations (for dietitians and their clients), lack of interest among potential clients in spending additional time required for assessment, and lack of incentives for potential clients. Time was extended for 1 location to increase participation, but because of the extended initial IRB review process, and because multiple IRBs were involved, others did not want to engage in an amendment process to extend the time for data gathering.

We did not identify whether the dietitians participating in the pilot study had educational materials targeting the spectrum of health literacy levels that were available for use in educational encounters. Lack of variability in educational materials makes it difficult to derive relationship between use of the NLAI and adjustment in teaching methods made by the dietitian as a result of the assessment. Additionally, although written materials are commonly used in nutrition education and were included in data assessment, oral instruction is an important method of education as well and is not investigated here.

Although most participants did not prefer the REALM over the NLAI, and most indicated they would use an assessment tool if it were available, we did not specifically ask whether the participants would use the NLAI. We interpreted the positive responses to the content of the NLAI and the minimal number of suggestions for additions to mean that the NLAI would serve their needs and they would use it. Further testing of the applicability in the clinical setting is warranted.

Another potential limitation is that we did not gather demographic information from our pilot participants. We did not compare age, socioeconomic status, race/ethnicity, or educational attainment of our sample with the characteristics of samples used to develop other instruments.

The lack of agreement between the subjective assessment and the NLAI in the pilot study suggests a need for more objective measures for determining nutrition literacy. Although the Newest Vital Sign has received some attention in nutrition literature (28,29) as an instrument that can reliably and quickly assess health literacy, and its use of the Nutrition Facts label may be preferable to other health literacy instruments for nutrition practice, our survey indicates that dietitians believe nutrition literacy requires skills and knowledge beyond an ability to read food labels. Additionally, testing of the Newest Vital Sign in an elderly African American population indicates its practicality may be limited (30).

Registered dietitians participating in our online survey found the NLAI to be content valid as a measure of nutrition literacy. Although participants gave numerous suggestions for instrument improvement, the reader should exercise caution in overemphasizing any suggestion because most participants agreed with the approach and methodologies of the NLAI. Future research should evaluate use of the NLAI in clinical settings.
